# Frontal Plane Knee Kinematics and Kinetics During Gait in Children and Youth with Achondroplasia—Correspondence with Static X-Ray Images and Relevance to Symptoms

**DOI:** 10.3390/children12010078

**Published:** 2025-01-10

**Authors:** Matthias Hösl, Antonia Thamm, Faik Kamel Afifi, Sean Nader

**Affiliations:** 1 Gait and Motion Analysis Laboratory, Schön Clinic Vogtareuth, 83569 Vogtareuth, Germany; athamm@schoen-klinik.de; 2 Institute for Transition, Rehabilitation and Palliation, Paracelsus Medical University Salzburg, 5020 Salzburg, Austria; 3 Division of Orthopaedic Surgery, Hospital for Sick Children, Toronto, ON M5G 1E8, Canada; faik.afifi@sickkids.ca; 4 Specialist Centre for Paediatric Orthopaedics, Neuroorthopaedics and Deformity Reconstruction, Schön Clinic Vogtareuth, 83569 Vogtareuth, Germany; snader@schoen-klinik.de

**Keywords:** achondroplasia, gait analysis, varus, valgus, knee, radiology

## Abstract

**Background**: Frontal knee malalignments are hallmarks of Achondroplasia (ACH), along with disproportional short stature. Typically, X-rays are used to assess them, but 3D gait analysis (3DGA) may additionally be used to evaluate dynamic knee function. The research questions were as follows: (1) What is the relationship between X-rays and 3DGA in ACH? (2) Do children with ACH have abnormal frontal knee kinematics and kinetics? (3) Are there aspects of 3DGA that relate to knee symptoms? **Methods**: A total of 62 knees of 31 children with ACH (age: 11.1 ± 4.3 years, 34 symptomatic knees) underwent 3DGA and X-ray as part of their standard clinical care. X-rays were analyzed for mechanical tibiofemoral angle (mTFA). Relationships between X-rays and 3DGA were determined. Sixty-two knees of 31 age-matched typically developing (TD) children served as references for 3DGA. Frontal knee kinematics (including thrust RoM) and adduction moments (KAMs) were compared. Multiple regression was performed for measurements associated with KAM, and ANOVA was used to compare TD and ACH knees with and without pain. **Results:** There was a high correlation between static frontal knee angles and mTFA (r = 0.93, *p* < 0.001, mean difference = −2.9°). ACH knees with a regular mTFA also showed significantly increased KAM. Multiple regression analysis showed that mTFA was the most relevant predictor of KAM (R^2^ = 0.41–0.75). Symptomatic knees (n = 34/62) experienced significantly more knee RoM in early stance than asymptomatic knees. **Conclusions:** Three-dimensional gait analysis may be an objective screening method for dynamic knee alignment and stability and may complement radiography in monitoring ACH. Symptoms may depend on knee thrust, while the impact of altered KAM needs further study.

## 1. Introduction

Achondroplasia (ACH) is a genetic disorder resulting in short-limb skeletal dysplasia [1]. It is caused by a mutation in the fibroblast growth factor receptor 3 (FGFR3) gene that results in impaired bone growth within cartilage, which leads to shorter long bones relative to the torso, with the upper parts of arms and legs being shorter than the lower portions (rhizomelic shortening) [2]. It is the most common form of disproportionate dwarfism, with a prevalence of 3.7–4.6 per 100,000 births [3,4].

Musculoskeletal abnormalities in ACH affect the spine, with narrowing of the thoracolumbar and lumbar canal and spinal stenosis or thoracolumbar kyphosis [5]. Concerning the muscles, hypotonia [6], a general decrease in strength [7], increased fat infiltration [8], and lower strength-to-muscle size ratios [9] were reported as potentially being further affected by altered neuromuscular coordination, e.g., higher antagonist co-activation [9] or complications of impinged nervous tissue [6,10]. Concerning the legs, frontal knee malalignments are particularly common [11]. Although pronounced genu varum was estimated to be present in ~60–7x% of patients [12], some knees may also deviate into significant valgus [13,14,15]. Various influencing factors are discussed, including pronounced medio-lateral instability, coxa vara, frontal plane tibia and femur deformities, internal tibial torsion, and recurvatum or fibular overgrowth [1,12,16,17,18]. Apart from altered bone geometry, mineral density and microarchitecture change with worse trabecular bone microarchitecture, thicker cortical bone thickness, and higher bone strength [19].

Among the most frequent impacts of ACH in the daily lives of children are difficulties walking long distances (71%) or generally being physically active (43%) [20]. Experts state that bowing of the legs can cause knee pain [21]. Potentially, a misaligned knee leads to unnatural movement, which may lead to overloading of joint structures, including ligaments, and cause further instability. This may cause pain and contribute to reduced walking distance; however, a clear link has not yet been established. Additionally, inconsistent reports on knee symptoms in childhood or the risk of knee osteoarthritis (OA) in adulthood in ACH exist [22]. On the one hand, knee and ankle musculoskeletal pain was reported to affect 70% of children with ACH [18]. On the other hand, joint replacement in adults is rare and degenerative changes are doubtful [23]. Notably, patients may also suffer from severe laxity [1,24]. Based on their clinical expertise, different authors have stated that knee pain in ACH may not be related to deformity but to hypermobility [1,12].

Since frontal knee deviations often appear to be pronounced in ACH, monitoring them is recommended. Hence, in orthopedics, standing weight-bearing antero-posterior radiographs are often performed. To reduce exposure to radiation, manual goniometry, measurements of distances between the knees, mid-tibiae, or malleoli, and visual plumb line assessments [1,11], as well as visual observation of thrust (uncontrolled lateral or medial knee movement), are advised [1]. However, such observations may not represent an objective evaluation.

Three-dimensional gait analysis (3DGA) may be more suitable. This analysis has already been used in patients with ACH [16,25,26,27]. Amongst others, patients generally walk slower and show a greater cadence [25,27]. Their flexed knees and dorsiflexed ankles are likely necessary to maximize toe clearance during swing [25]. Also, an increased anterior pelvic tilt seems to be a consistent finding [25,26,27]. For the knee, some reported no differences in frontal kinematics between ACH and controls [26], while others reported characteristic varus alignments (leg bowing) during gait [16,27]. According to the assumptions of Hoover-Fong and associates [28], most knee deformities in ACH may not actually depend on static bony malalignment but on lateral thrust during gait due to laxity. In osteoarthritis (OA), varus thrust was associated with radiographic pathology [29] and greater odds for OA progression [30].

In order to use 3DGA more extensively in ACH, it is also important to know whether the radiological pathology can be mapped with motion capturing. For idiopathic lower limb malalignment, the mechanical tibiofemoral angle (mTFA) from X-rays highly correlated (r = 0.81) with the marker-based knee angle [31]. Also, in children with Pseudo-Achondroplasia, a diverse group of skeletal dysplasia and rhizomelic dwarfism, a moderately strong correlation (r = 0.70) between radiographic axes of the femur and tibia and the frontal plane knee angles while walking was found [32]. On the other hand, a poor correlation with measures of genu varum during gait and X-rays in ACH was reported [16]. This has not been disproved.

Apart from knee kinematics, joint moments from 3DGA are often used as a surrogate for medial-lateral knee load distribution. The knee adduction moment (KAM) is focused in patients with normal stature. Based on the KAM, children with idiopathic varus-valgus knees show altered loading of the medial or lateral compartments [33,34,35]. There are also typical gait compensations to lower the KAM, for example, trunk lean, wider steps, or altered foot progression angle [36,37,38]. There have so far been inconsistent reports about alterations in KAM in ACH [16,27]. Finally, the research group led by Broström and associates [27] suggested that future research should focus on the relationship between gait and physical symptoms. Thus, the aims of the current study were as follows:

(1) Quantify the agreement between X-rays and standing motion capture in ACH.
(2) Test whether children with ACH have abnormal knee kinematics and kinetics in the frontal plane during gait. Herein, investigate how well radiographic knee malalignments actually predict KAM while walking and whether this relationship is modified by gait compensations.
(3) Investigate whether knee kinematics and KAM alterations are associated with symptoms.

We hypothesized that there is a significant relationship between radiography and knee kinematics and the KAM. We expected that walking may aggravate static malalignments and feature increased knee thrust in ACH with respect to TD, as well as altered KAM, in particular in ACH patients with knee symptoms.

## 2. Materials and Methods

### 2.1. Participants

We retrospectively screened a database of a clinical gait laboratory for patients diagnosed with ACH who had 3DGA, including valid kinematics and kinetics, as part of their clinical routine. We then searched for those who might have had a frontal plane long leg X-ray within close temporal proximity (at most 4 months before or after). Further exclusion criteria were any leg surgeries within the last 6 months, previous leg lengthening procedures, and age below 6 years. This retrospective study was approved by the Ethic Commission of LMU Munich (Vote Nr. 21-0617) and was exempted from further written consent due to the secondary analysis of existing and anonymized data. A total of 31 children and youth with ACH (11.1 ± 4.3 y. [6–19]) from a clinical gait laboratory database were retrospectively included in the study. Thirty-one TD (11.7 ± 3.9 y.) were matched from our normative controls and served as a reference during gait analysis. Data for both legs were included. Age, height, weight, and history of 8-plates were extracted from patient records (Table 1). Concerning pain history, patients were given a questionnaire in the course of 3DGA to assess pain in everyday life using a body map [39]. ACH knees were categorized into pain/no pain, depending on whether symptoms were reported.

### 2.2. X-Rays

Standing radiographs had been taken with X-rays pointing perpendicular to the femoral condyles on a full-length radiograph. We selected the hip–knee angle (HKA) representing the varus/valgus configuration. A value > 0° represents a varus alignment, and a value < 0° represents a valgus alignment. The HKA is here referred to as the mechanical tibiofemoral angle (mTFA).

### 2.3. Three-Dimensional Gait Analysis (3DGA)

TD and ACH patients walked barefoot overground at a self-selected speed in a gait laboratory. Kinematic data were collected using a 12-camera Vicon Vero motion capture system (VICON Motion Systems, Oxford, UK) operating at 200 Hz, while two AMTI OPTIMA force plates (Advanced Mechanical Technology, Inc., Watertown, MA, USA) were used to synchronously collect kinetic data at 1000 Hz. The marker protocol was a modified version of the Plug-in-gait model. Knee and ankle joint axes were defined by a medial and lateral marker on the epicondyle of the femur and the medial/lateral tip of the malleolus. One additional marker was applied to the skin over the greater trochanter, and the a-p component of the ASIS-Trochanter distance was determined in the local coordinate system of the pelvis segment. Leg length was additionally extracted based on markers from standing trials, calculated as the sum from the anterior iliac spine to the center of the knee joint and from the center of the knee joint to the center of the ankle joint.

### 2.4. Data Analysis

Given reference data from TD children with normal height [40], an mTFA alignment of ≤±3° of valgus or varus was conservatively considered to be a regular mTFA, and ACH knees were categorized into valgus, varus, or normal alignment.

During 3DGA, at least 5 gait cycles per leg were extracted and averaged for kinematics and kinetics. To quantify the static alignment, the standing trial from 3DGA was used. Concerning KAM, non-dimensional normalization was used to account for body weight (mass [kg] × 9.81 [m/s^2^]) and leg length [m]; thus, KAM was expressed in Nm/(BW × LL) [41]. The maximum knee adduction moment during the first (KAM-1) and second half of stance (KAM-2) was determined for each trial and averaged. In addition, the minimal KAM was extracted during early stance (KAM-0), and the average during single stance was calculated (KAM_midstance_).

To quantify weight-bearing knee thrust, we extracted the valgus–varus knee RoM from IC (initial contact) until midstance (1/2 stance phase) [42]. In addition, we extracted the foot progression angle (mean value in single stance), the ipsilateral trunk lean [min value in single stance], the hip rotation (mean value in single stance), and step width as potential factors that influence the KAM.

Finally, walking speed, cadence, and step length were extracted and taken as absolute values but also non-dimensionally normalized [43].

### 2.5. Statistics

General demographics and patient characteristics between ACH and TD were compared using independent *t*-tests. Gender distribution and the proportion of painful knees in ACH valgus, ACH norm, and ACH varus knees were compared using the Chi-squared test. To assess the relationship between 3DGA and X-rays, linear regression was performed between mTFA and standing frontal plane knee angles, as well as between mTFA and the average knee angle during single stance.

Statistical parametric mapping was used to compare frontal plane knee angles and the KAM between TD and ACH knees with valgus, varus, and normal mTFA alignment, as well as to compare ACH knees with and without pain. Mean curves between groups were compared using a *t*-test within SPM1-D [44].

In order to predict the KAM, we performed 4 multiple regressions using the mTFA from X-ray as well as the foot progression angle, the hip rotation, the ipsilateral trunk lean, and step width as predictors for KAM-0, KAM-1, KAM_midstance_, and KAM-2. The calculations were carried out using the function “stepwisefit” in the MATLAB statistics toolbox. The maximum *p*-value for a term to be added was *p* < 0.05. The minimum *p*-value for a term to be removed was *p* > 0.10. To compare ACH knees with and without pain and knees of TD controls, we performed a one-way between-subjects ANOVA with subsequent post-hoc tests on tempo-spatial parameters, as well as on kinematic knee parameters of knee thrust and on kinematic gait adaptation values. All analyses were conducted in MATLAB R2022b. Due to the explorative nature of the study, the alpha value was set to 0.05.

## 3. Results

### 3.1. Patient Characteristics

Table 1 shows that patients with ACH were smaller in height (*p* = 0.027) and had shorter legs (*p* < 0.001) and higher BMI (*p* < 0.001). Gender distribution was not different between ACH and TD. Overall, 34 of 62 (54.8%) knees with ACH presented with symptoms (Table 1). A total of 15 of 31 ACH patients had bilateral symptoms, four unilaterally. Age, BMI, and leg length (*p* = 0.471–0.770) were not different between ACH knees with and without pain.

### 3.2. X-Rays

When categorized according to radiographic mTFA, 62.9% (39 of 62) of the ACH knees showed a varus deviation > 3°, which averaged 12.4 ± 7.9°, and 16.1% deviated into valgus < −3° (10 of 62, mTFA: −11.0 ± 13.3°). The alignment of ACH normal (n = 13) was 0.17 ± 1.77°. The average time span to 3DGA was 0.8 ± 1.3 months, with a maximum of 4 months.

### 3.3. Knee Joint Kinematics

**Figure 1:**
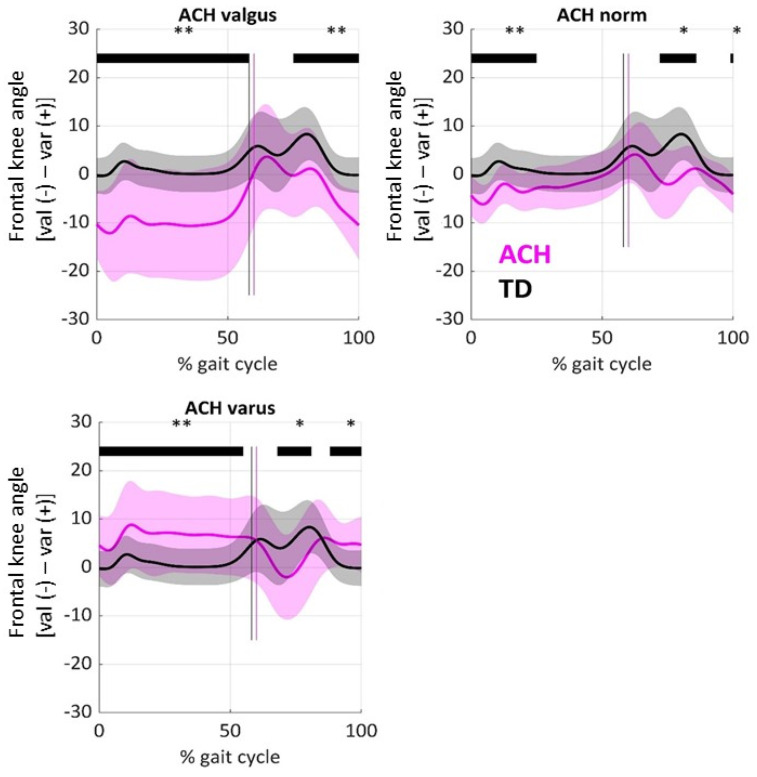
Frontal plane knee angle in patients with ACH (pink) and in typically developing (TD) controls (grey). The mean (line) and standard deviation (shaded area) are displayed. ACH knees were categorized according to X-ray alignment of the mTFA. Vertical lines mark the end of the stance phase. Horizontal bars mark significant differences from TD controls based on Statistical Parametric Mapping (* *p* < 0.05, ** *p* < 0.01).

As expected, Figure 1 shows that there was significantly more valgus throughout the stance in radiographic ACH valgus knees and more varus in ACH varus knees. Apart from this, knees with a rather regular mTFA (ACH norm) showed increased valgus during the initial stance, which shifted towards the varus direction and corrected until midstance.

### 3.4. Relationship Between X-Ray and Knee Kinematics

**Figure 2:**
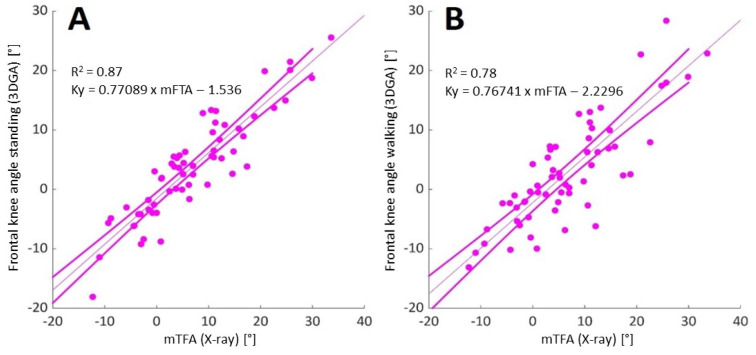
Linear regression (95% confidence interval) between radiological mechanical tibiofemoral angle (mTFA) and frontal plane knee angle (Ky) (**A**) during static bipedal standing and (**B**) during walking (single stance phase) in patients with Achondroplasia (negative values~valgus).

Figure 2A shows that there was a strong linear relationship between mTFA from X-ray and bipedal standing frontal plane angles in ACH (r = 0.93, *p* < 0.01), with a mean difference between methods of −2.9 ± 4.5°. Figure 2B displays a decrease in the strength of the relationship during the single stance phase of walking (r = 0.88, *p* < 0.01).

### 3.5. Knee Adduction Joint Moments (KAMs)

**Figure 3:**
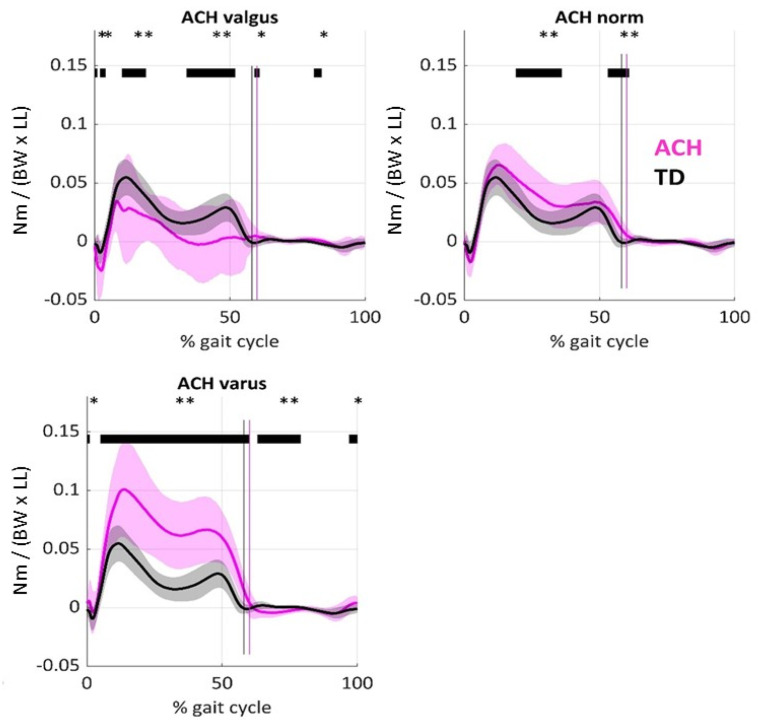
Knee adduction moments (KAMs) in patients with ACH (pink) and in typically developing controls (grey). The mean (line) and standard deviation (shaded area) are displayed. ACH knees were categorized according to X-ray alignment of the mTFA. Vertical lines mark the end of the stance phase. Joint moments were non-dimensionally normalized by body mass (unit: Nm/(kg × 9.81 m/s^2^ × leg length [m])). Horizontal bars mark significant differences from controls based on Statistical Parametric Mapping. ** *p* < 0.01, * *p* < 0.05.

Concerning SPM1D analysis, we observed significantly decreased KAM in ACH valgus knees with respect to TD (Figure 3), most pronounced during the second half of stance, while in ACH knees with varus alignments, the KAM was significantly increased throughout most parts of stance. In ACH knees with regular radiological alignments, we observed a significantly increased KAM in single stance, as well as during terminal stance.

### 3.6. Relationship Between X-Ray, KAM, and Gait Compensations

Knee adduction moments (KAMs) were predicted by mTFA (Table 2), with the explained variance ranging between 41.2 and 74.7%. Foot progression angle (KAM-0, KAM_midstance_), ipsilateral trunk lean (KAM-1), and hip rotation (KAM-2) modified the magnitude of KAMs, explaining about 2–4% of further variance.

### 3.7. Comparisons of Knees with and Without Pain in Achondroplasia

#### 3.7.1. X-Rays

The prevalence of pain was 40% for ACH valgus (four of ten), 56.4% (22 of 39) in ACH varus, and 61.5% (8 of 13) in ACH knees with regular mTFA. Chi-square test indicated that the probability of pain was not different between groups with different knee alignments (*p* = 0.559). Also, mTFA was not different between ACH knees with and without pain (6.4 ± 12.7° vs. 5.5 ± 11.6°, *p* = 0.762). Four of eleven knees with current 8-plates reported pain (36.3%).

#### 3.7.2. Tempo-Spatial Parameters

In absolute terms, patients with ACH walked slower than TD controls (both *p* < 0.001) (Table 3); however, ANOVA failed to indicate differences between ACH and TD patients concerning normalized gait velocity (*p* = 0.058). Absolute cadence was increased, on average, by 14–15 steps/min in ACH knees with and without pain (both *p* ≤ 0.001), while step length was reduced (both *p* < 0.001). When normalized to leg length, cadence was lower in ACH, while relative step length was increased (*p* < 0.001), and the increase in relative step length was larger in ACH knees with pain (*p* = 0.011).

#### 3.7.3. Knee Joint Kinematics

**Figure 4:**
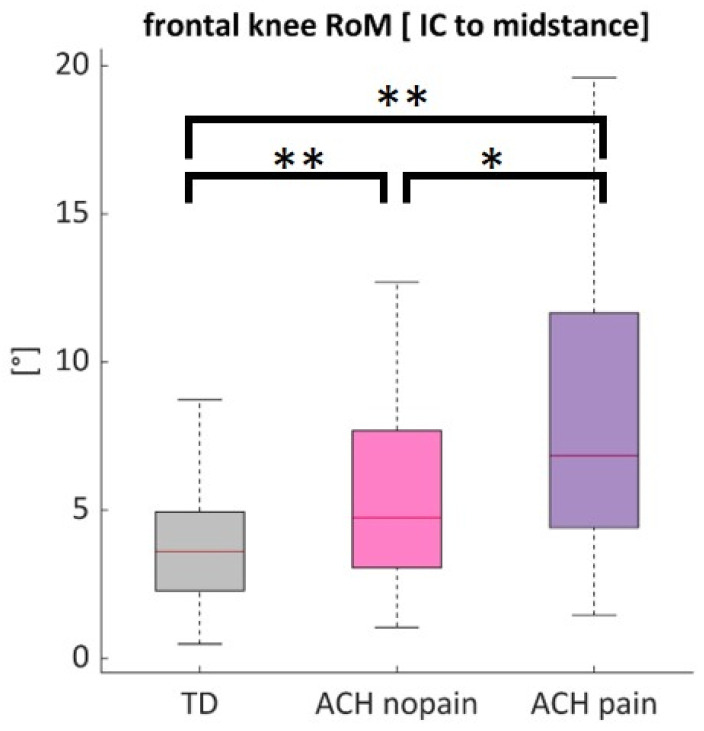
Knee valgus–varus RoM upon weight bearing from initial contact [IC] to midstance during gait in typically developing (TD) knees (grey), ACH knees without pain (pink), and ACH knees with pain (purple). ** *p* < 0.01, * *p* < 0.05.

SPM1D revealed no significant differences between ACH knees with pain and ACH knees without pain across the gait cycle. ACH knees with pain showed significantly more frontal plane knee RoM from initial contact until midstance than ACH knees without pain (*p* = 0.014) and TD (*p* < 0.001) (Figure 4).

#### 3.7.4. Knee Adduction Moments [KAMs] and Kinematic Gait Compensations

**Figure 5:**
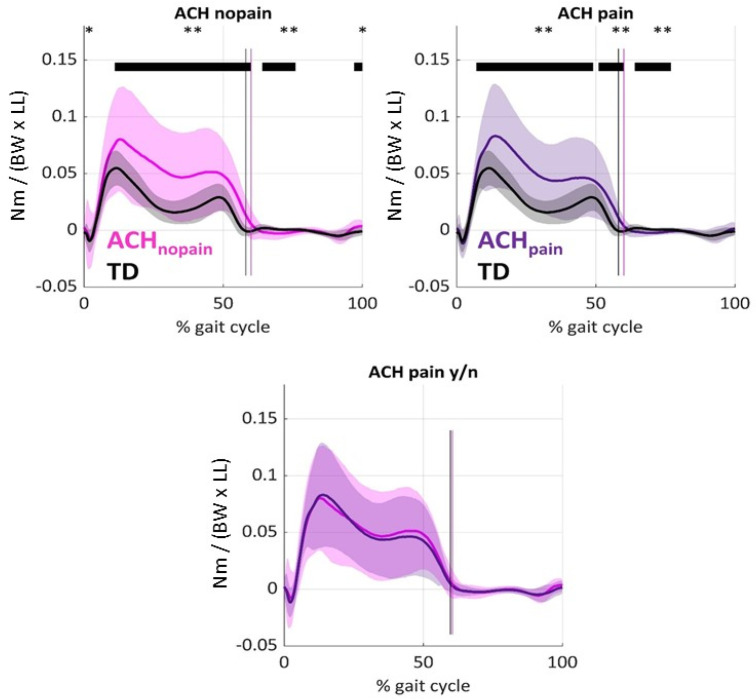
Knee adduction moments during gait. The mean (line) and standard deviation (shaded area) are displayed in typically developing knees (grey), as well as in ACH knees without (pink) and ACH knees with pain (purple). Vertical lines mark the end of the stance phase. KAMs were normalized (Nm/(body mass [kg] × 9.81 [m/s^2^] × leg length [m])). ** *p* < 0.01, * *p* < 0.05. Horizontal bars mark significant differences from TD. NB: No significant differences were found between ACH knees with and without pain.

**Figure 6:**
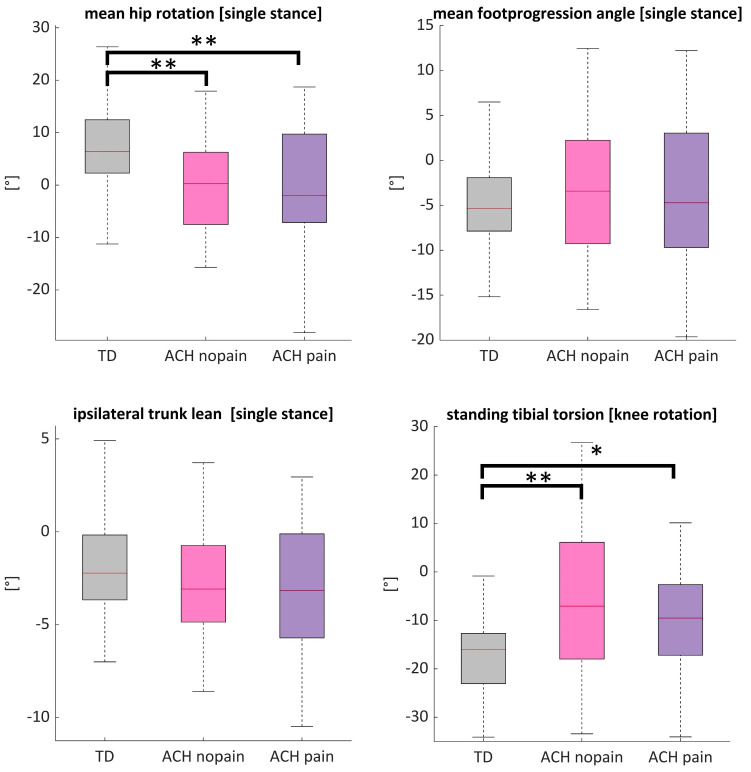
Kinematic gait compensations that might affect the KAM. ACH knees with pain (purple) and without pain (pink) vs. TD (grey). Lower right graph: standing knee rotation as a proxy for tibial torsion. ** *p* < 0.01, * *p* < 0.05.

On average, KAM in both ACH pain and ACH no pain were significantly increased with respect to TD (Figure 5). Figure 6 shows that ACH knees with and without pain showed more external hip rotation (both *p* ≤ 0.009) and less external rotation of the knee than TD (both *p* ≤ 0.033). ANOVA further showed no significant differences in foot progression angle (*p* = 0.791) or ipsilateral trunk lean (*p* = 0.233) between groups.

## 4. Discussion

This study had three main goals: first, to quantify the agreement between X-rays and motion capture in Achondroplasia (ACH); second, to assess whether children with ACH show abnormal frontal knee kinematics and kinetics and whether radiographic malalignment can predict abnormal knee adduction moments (KAMs); third, to explore whether changes in knee kinematics and KAM are linked to symptoms. The main findings were a strong relationship between frontal knee angles and mTFA. While mTFA deviations also predicted KAM deviations during gait, even knees with regular mTFA had a higher KAM than TD children. Symptomatic knees were characterized by higher medio-lateral knee thrust, not by increased KAM; thus, 3DGA is valid and useful for assessing knee alignment and stability in ACH. Our findings emphasize the need to focus not only on static alignment but also on dynamic knee function. Practically, it can be integrated into routine check-ups with pediatricians and orthopedists, offering a non-invasive way to monitor knee function and detect abnormal dynamics early, guiding personalized interventions. Theoretically, this enhances our understanding of the patho-biomechanics in ACH, particularly abnormal frontal knee thrust. The results could motivate future research on the underlying unfavorable factors influencing these movements.

### 4.1. Relationship Between X-Ray and 3DGA in Achondroplasia

The strong relationship (r = 0.93, *p* < 0.01) emphasizes the potential of 3DGA as a non-invasive monitoring tool for ACH. Inan and colleagues [16] previously found no correlation between patients with ACH and genu varum. Our high correlation could be due to the larger sample size, a different marker protocol, the dispersion of malalignments (varus and valgus knees in the current ACH cohort), and our focus on standing vs. walking. Figure 2B also shows that the relationship between X-ray and dynamic knee alignment during single stance was fairly high, even though it was lower than during bipedal standing. The average offset to the mTFA during standing appears larger in ACH patients than in idiopathic knee deformities: −2.9° (current data) vs. −1.8° [31]. Both indicate a shift to valgus. The larger inconsistency in ACH knees may be attributable to pose variations, projection errors in X-rays, or under-predicted varus due to hip center misallocation. Several authors have noted that hip center allocation might affect the frontal knee angles in ACH [26,27,45], as the radiological mechanical axis of the femur may not coincide with the primary vertical femur axis from 3DGA. It is worth noting that the PiG model relies on anthropometric-based regression equations to estimate the hip joint center [46]. Broström and associates [47] used functional standing star calibration [48] on N = 4 patients with ACH; this should be tested in larger cohorts.

### 4.2. Gait Abnormalities in Knee Kinematics and Kinetics in Achondroplasia

The 3DGA further revealed dynamic knee instability (more frontal RoM) and altered KAM in ACH patients, which partly reflected their underlying bony malalignments (mTFA). However, even knees with a fairly regular mTFA (within ±3° of neutral) displayed a significantly higher KAM than TD, often used as a surrogate for increased joint load. Naili and associates [49] reported a relationship between KAM and structural findings of knee OA severity in adult OA patients. Any gait-model-related underestimation of knee varus angle might have further underestimated this increase in KAM. Focusing solely on X-rays for decision-making may miss this. Broström and associates [27] did not observe alterations in KAM in ACH despite showing significantly more knee varus than TD. In particular, collapsing both varus and valgus deviating knees could likely blur cross-sectional differences. The aforementioned group [27] further reported that patients with ACH show pronounced outward rotation of the hip throughout stance. In the current sample, more external hip rotation than TD was confirmed. According to the regression, rotating the hip externally will increase KAM-2 and could thus be a negative factor for knee loads in varus deviating knees.

In this study, mTFA explained ~41 to 74% of the variance in KAM, making it the most important factor influencing altered knee loads in ACH. Correlations between KAM and mTFA were previously found to be 0.51–0.61 in adult OA patients [37,38,50]. Our multiple regression further suggested that the KAM can, to some degree, be modified in ACH. Modification of the foot progression angle has also been shown to alter the point of application of the ground reaction force, while trunk lean shifts the center of mass closer to the knee joint center serving as an unloading strategy, similar to patients with medial knee OA [37]; however, the explained variance was lower. It seems that the usual compensation for modifying the KAM is less effective in ACH than in people of normal stature. Modification in the foot progression angle modifies the KAM-2, indicating further increase with internal foot orientation [38]. This might also be influenced by reduced external torsion of the tibia, which is typical in ACH and also confirmed during our standing trial (Figure 6). Other studies further revealed that the KAM depends on the transversal knee rotation [51,52]. To avoid statistical overfitting, we did not include the knee rotation in the model.

### 4.3. Factors Related to Knee Pain in Achondroplasia

At first sight, the proportion of painful ACH knees appears large, but this confirms previous reports [18]. Knee pain may also potentially contribute to the limited walking distance reported in ACH [20]. Symptomatic knees exhibited more frontal RoM (likely uncontrolled varus–valgus motion upon weight bearing) than asymptomatic knees. To the best of our knowledge, this is the first study to show that such medio-lateral thrust parameters relate to knee symptoms in ACH. Our results might further confirm a previous expert statement that knee pain in ACH is not primarily related to deformity but to hypermobility [12]. In older OA patients, varus thrust is associated with greater odds of OA progression [30], worsening of bone marrow lesions, tibiofemoral cartilage loss [53], and greater odds of pain [54]. The most likely mechanism by which thrust contributes to the progression of knee OA is through an increase in medial or lateral knee compartment loading; thus, thrust in symptomatic knees in children with ACH is thought provoking. Varus thrust has also been reported in some otherwise TD children aged 3–9 (41%) and adolescents 10–22 years (25%) [55]. The clinical significance of such thrust early in life is not known, and future research should determine whether it is detrimental to joint health in ACH.

Potential reasons for the thrust could be weakness, laxity, or anatomic deviations. Lower knee extensor and flexor strength are related to greater varus thrust in adults with OA [56]. We did not conduct similar instrumented strength tests in ACH; however, based on the manual and clinical examinations, the ACH groups did not differ. Using more sophisticated equipment, patients with ACH showed lower normalized force of the vastus lateralis compared with TD patients [45]. Additionally, general laxity could be critical, as children with ACH generally show a two times higher risk of ligamentous laxity than TD children, and about every second child with ACH may meet the diagnostic criteria for ligamentous laxity [57]. Ligamentous laxity was also previously associated with chronic pain in children with idiopathic laxity, likely due to repeated distortive traumas. Improving dynamic muscle control to supplement ligamentous insufficiency may minimize that in TD children [58]. In ACH, frontal plane laxity was reportedly caused by specific anatomic alterations as well as constitutional laxity, which could reduce tension in collateral ligaments [59]. Knees of ACH patients also have characteristic anatomy, e.i., a deep trochlea, a high intercondylar notch, a vertically oriented anterior cruciate ligament [24], a small patellofemoral groove [60], discoid lateral meniscus, often with a tear [61], and abnormal anterior slope of tibial plateaus [62], which could also disturb knee stability.

In contrast to knee thrust, KAM deviations could not be directly linked to symptoms in ACH knees. Our results suggest that abnormally high KAMs may, at first sight, not be decisive in ACH; however, taking their high cadence into account, the differences in KAM will be further aggravated when calculated as cumulative deviations when covering similar distances to a TD person. We suggest that chronically altered KAM may potentially contribute to progressive instability, and symptoms may develop if the increased KAM causes soft tissue structures and collateral ligaments to lose elasticity, which may subsequently result in increased medio-lateral thrust. Finally, the significantly longer relative step length in patients appears to be a form of overcompensation in ACH knees, which could predispose to knee pain and instability; however, the exact mechanism warrants further research.

### 4.4. Limitations and Recommendations

The authors of this study emphasize that the obtained results should be interpreted with certain limitations:

- First, all patients were from a pediatric orthopedic department sent for clinical evaluations. High proportions of symptoms and malalignments may not be generalizable to all children with ACH.
- Second, from a methodological perspective, the gait model could be further improved. Knee anatomy in ACH is special, and the PiG model might be a simplification of this. Also, imaging technology might be used in ACH to locate the hip joint centers relative to pelvis geometry, which might be altered in ACH in comparison to TD and assist in establishing disease-specific regression equations. As foot and ankle biomechanics can also have an impact on knee biomechanics, e.g., increased rearfoot inversion, which is typical in ACH and generally increases both KAM [63] and varus thrust [64], this argues for a more specific foot model for a detailed assessment.
- Third, concerning the X-rays, we focused on the mTFA only. The assessment of skeletal abnormalities and indications for possible intervention in ACH in relation to their axial abnormalities (e.g., 8-plates) includes significantly more X-ray parameters [13] than reported in the current study. To accurately guide orthopedic decision-making, the radiographic parameters that predispose to laxity need to be determined, as several joint orientation angles are usually taken into consideration when deciding on treatment; in particular, the interaction between these joint orientation angles and dynamic instability during gait should be investigated. In the future, a better understanding of the development of symptoms will likely be achieved by assessing the relationship between traditional imaging data, detailed clinical examinations, motion capturing as part of functional instrumented tests, and, most importantly, patient-reported outcomes in longitudinal follow-up studies of ACH patients throughout their maturation.

## 5. Conclusions

Knee symptoms in ACH may depend on valgus–varus thrust RoM during gait. The significance of altered KAM and related gait modifications warrants further studies. Motion capturing may function as an objective screening tool for knee malalignment and for dynamic instability during gait in children and youth with ACH. This is fairly relevant to patients and parents, as it may assist in reducing radiation exposure, e.g., when monitoring growth modulation by tension band plates or assessing the functional impact of new growth-promoting drugs. In the future, 3DGA could be part of regular health supervision in ACH and could complement radiography for orthopedic decision-making. Due to the complex anatomy, motion capturing may not currently be a full surrogate for radiography.

## Figures and Tables

**Table 1:** Patient characteristics in typically developing (TD) controls and in children with Achondroplasia (ACH).

	TD (n = 31, 62 Knees)		ACH (n = 31, 62 Knees)		
	Mean	SD	Mean	SD	*p*-Value
Age (years)	11.1	4.3	11.7	3.9	0.606
Height (cm)	145.9	24.5	112.9	13.9	0.027
Leg length (cm)	76.5	14.5	44.7	7.0	<0.001
Weight (kg)	39.1	17.7	31.7	11.1	<0.001
BMI (kg/m^2^)	17.3	2.4	24.0	3.9	<0.001
Gender [Nr of sub.]—[m/f]	14/17		13/18		0.797
Pain [Nr of knees]—[y/n]	0/62		34/28		<0.001
8-Plates [Nr of knees] (current/previous/none)	0		11/8/43		n.a.

Mean, standard deviation (SD), and the *p*-value of the group comparisons; n = nr of subjects; n.a. = not applicable.

**Table 2:** Results from multivariate regression on the knee adduction moment (KAM) during gait in participants with Achondroplasia (ACH).

**Prediction of KAM-0**				
**Predictor Variables**	**β**	***p***	**SE**	**Δ Adj. R²**
mTFA [°] (v1)	0.000846	<0.001	0.000201	0.412
foot progression angle [°] (v2)	0.000663	0.024	0.000286	0.039
**Prediction equation**				**Total R²**
KAM-0 = −0.0181 + 0.000846 × v1 + 0.000663 × v2				0.451
**Prediction of KAM-1**				
mTFA [°] (v1)	0.002682	<0.001	0.000292	0.548
ipsilateral trunk lean [°] (v2)	0.002333	0.032	0.001063	0.027
**Prediction equation**				**Total R²**
KAM-1 = 0.080071 + 0.002682 × v1 + 0.002333 × v2				0.575
**Prediction of KAM_midstance_**				
mTFA [°] (v1)	0.002133	<0.001	0.000553	0.747
Foot progession angle [°] (v3)	0.000869	0.006	0.006117	0.026
**Prediction equation**				**Total R²**
KAM_midstance_ = 0.04737 + 0.002133 × v1 + 0.000869 × v2				0.773
**Prediction of KAM-2**				
mTFA [°] (v1)	0.002345	<0.001	0.000203	0.671
Hip rotation [°] (v2)	−0.000455	0.025	0.000197	0.022
**Prediction equation**				**Total R²**
KAM-2 = 0.043897 + 0.002345 × v1 − 0.000455 × v2				0.693

KAM-0 = minimal moment during early stance, KAM-1 = maximal moment during the first half of stance, KAM-2 = maximal moment during the second half of stance, β = the standardized regression coefficient, SE = Standard error, adjusted R^2^ = explained variance, Δ adjusted R^2^ = increase when adding this variable to the model.

**Table 3:** Comparisons of tempo-spatial parameters in patients with Achondroplasia (ACH) and typically developing (TD) controls with and without knee pain. Upper part: absolute values; lower part: normalized as non-dimensional numbers and scaled quantities.

	TD		ACH _no pain_		ACH _pain_		ANOVA
	M	SD	M	SD	M	SD	*p*-Value
v (m/s)	1.25	0.14	0.95	0.13 **	0.97	0.11 **	<0.001
cadence (steps/min)	129.05	19.03	144.72	20.42 **	143.63	15.26 **	<0.001
step length (m)	0.58	0.10	0.39	0.06 **	0.40	0.06 **	<0.001
non-dim velocity	0.46	0.06	0.46	0.06	0.47	0.05	0.058
non-dim cadence	0.59	0.04	0.51	0.05 **	0.51	0.03 **	<0.001
norm step length [% LL]	76.57	7.13	88.98	8.20 **	90.31	9.94 **^,†^	<0.001

** *p* < 0.01 ACH vs. TD. ^†^ *p* < 0.05 ACH no pain vs. ACH pain. non-dimensional: normalization in which measurements are rendered dimensionless. LL: leg length.

## Data Availability

The data are not publicly available due to restrictions, e.g., privacy or ethical restrictions. The data are available from the corresponding author on reasonable request.
